# Venous Thromboembolism Risk in Hematological Malignancies Post-Chimeric Antigen Receptor T-Cell (CAR-T) Therapy: A Meta-Analysis of Phase 2 and Phase 3 Clinical Trials

**DOI:** 10.3390/curroncol31080323

**Published:** 2024-07-30

**Authors:** Akshit Chitkara, Sushanth Sreenivasan, Yue Yin, Maitreyee Rai, Santhosh Sadashiv

**Affiliations:** 1 Department of Internal Medicine, University of California, Riverside, CA 92521, USA; akshit.chitkara@jefferson.edu; 2 Department of Internal Medicine, Allegheny Health Consortium, Pittsburgh, PA 15222, USA; 3 Allegheny Singer Research Institute, Pittsburgh, PA 15212, USA; yue.yin@ahn.org; 4 Division of Hematology and Cellular Therapy, Allegheny Health Network Cancer Institute, Pittsburgh, PA 15224, USAsanthosh.sadashiv@ahn.org (S.S.)

**Keywords:** Chimeric Antigen Receptor T-cell (CAR-T), venous thromboembolism, meta-analysis, hematological malignancies, mantle cell lymphoma, B-cell lymphoma, multiple myeloma

## Abstract

Chimeric Antigen Receptor T-cell (CAR-T) therapy uses genetically engineered T-cells with specific binding sites. This therapy allows for tumor specificity and durable treatment responses for patients with hematological malignancies. In this review, we study the risk of venous thromboembolism (VTE) associated with CAR-T therapy. We searched the National Institutes of Health library, Cochrane Library Databases, ClinicalTrials.gov database, and medical literature search engines PubMed and Google Scholar for Phase 2 and Phase 3 drug-efficacy and safety trials to determine the aggregate incidence and risk of VTE treated with CAR-T. Of 1127 search results, nine studies were identified and included in our meta-analysis. Of the 1017 patients who received therapy, 805 patients (79.15%) experienced some degree of CRS, and 122 patients (11.9%) experienced severe CRS (higher than grade 3). Only three out of one thousand and seventeen patients were reported to have experienced venous thromboembolism. Our study did not find a statistically significant association between increased VTE incidence (OR = 0.0005, 95% CI [0.0001, 0.0017]) and CRS/ICANS (*p* < 0.0001). There was a 0.0050 (95% confidence interval [0.0019, 0.0132]) relative risk for VTE. In our study, we did not find a statistically significantly increased risk of developing VTE despite CRS and underlying malignancy, which have been associated with increased risk of VTE.

## 1. Introduction

Chimeric Antigen Receptor T-cell (CAR-T) therapy uses genetically engineered T-cells that have a specific antigen binding site to tumor cells, which allows for tumor specificity and durable treatment responses for patients with hematological malignancies. As of 2022, there are six FDA approved CAR-T therapies available for B-cell leukemias and lymphomas [1] and two approved for multiple myeloma [2,3]. However, through clinical trials, CAR-T therapy continues to expand as a treatment option for hematological malignancies and solid tumors. In this review, we study the risk of venous thromboembolism (VTE) during CAR-T therapy.

CAR-T is associated with two life threatening cytokine-associated adverse effects: immune effector cell-associated neurotoxicity syndrome (ICANS) and cytokine release syndrome (CRS) [4]. Cytokine-associated toxicity is a non-antigen-specific toxicity caused by high-level immune activation through myeloid and T-cells [4]. CRS is a clinical syndrome characterized by high levels of inflammation, and clinically manifesting as fever, hypotension, and hypoxia [5]. In theory, the high levels of inflammation should increase the patient’s risk of venous thromboembolism (VTE). It has been proposed that inflammation of the vessel wall initiates thrombus formation in an intact vein via coupling of the inflammatory and coagulation systems [6].

There are only a few case reports and retrospective analyses that have described the risk of VTE after CAR-T therapy in patients with hematological malignancies [7,8,9,10]. In this systematic review and meta-analysis, we reviewed data from Phase 2 and Phase 3 B-cell targeted CAR-T drug trials that primarily analyzed drug-efficacy and safety, in order to determine the aggregate incidence and risk of VTE post-CAR-T treatment in patients with hematological malignancies. 

## 2. Materials and Methods

### 2.1. Study Search and Selection

We sought to extrapolate the observed incidence of VTE in adult patients with active hematological malignancies who underwent CAR-T therapy from large Phase 2 and Phase 3 clinical drug trials. Completed Phase 2 and Phase 3 drug-efficacy trials report all adverse effects that occur during the follow-up phase and thus were ideal for our meta-analysis. A search was conducted, without language restrictions, of the National Institutes of Health library, Cochrane Library Databases, ClinicalTrials.gov database, and medical literature search engines PubMed and Google Scholar for all CAR-T drug-efficacy trials that were initiated after January 2014 and completed by 7 March 2023 and tested drugs used in hematological malignancies. The following search terms were used to narrow the search: chimeric antigen receptor T-cell, CAR-T therapy, FDA approved, venous thromboembolism, VTE, pulmonary embolism, Phase 2 and Phase 3. Boolean operators (“and”, “or”, and “not”) were used in conjunction with the search terms to target the relevant studies. 

Two reviewers independently reviewed the full texts of the selected articles and studies for inclusion and verified them for data extraction. Any disagreement was resolved with mutual discussion and the inclusion of a third (impartial senior) investigator. To be included, studies were required to be a Phase 1b/2 or Phase 3 clinical trial, be a completed trial with published results, have complete clinical data available, be focused on patients with active hematological malignancy, and have a comprehensive list of adverse effects. Studies or articles were excluded if they were single-center observational studies, incomplete studies, early Phase 1 trials, review articles, or works focused on non-hematological malignancies. 

### 2.2. Data Extraction

Data from these studies were extracted, verified, and analyzed by the authors of the study. We specifically evaluated the published and supplemental data for the incidence, onset, and duration of CRS and the incidence of VTE events. As the listed studies were clinical trials, they were obligated to list any adverse effects they encountered during the patient follow-up period. A data extraction form was utilized to collect the following information: therapy name, clinical trial phase, total enrolled patients, patients treated with CAR-T, CRS associated with CAR-T therapy, Grade 3–4 CRS associated with CAR-T therapy, median time of onset of CRS, median duration of CRS, VTE observed events, and the control group for VTE, if listed. The control group was defined as the patients who did not receive CART therapy and who developed VTE. We assumed that no VTE events occurred if there were none listed within the data set or supplementary information. The number of VTE events observed in the total number of treated patients was used in our calculations for odds ratio (OR) and relative risk.

### 2.3. Statistical Analysis

A biostatistician conducted the meta-analysis, using the Meta package in R version 4.1.2 [11]. The endpoints of interest were the OR and relative risk of developing VTE while in CRS. However, due to the sparsity of VTE events, a fixed-effect meta-analysis, specifically the Mantel–Haenszel method, was used to calculate the OR and relative risk. 

The I^2^ statistic was used to calculate heterogeneity: I^2^ < 25 was considered a low level of heterogeneity, an I^2^ value between 25–50 was considered a moderate level of heterogeneity, and any I^2^ value greater than 50% was considered a high level of heterogeneity. 

## 3. Results

**Figure 1:**
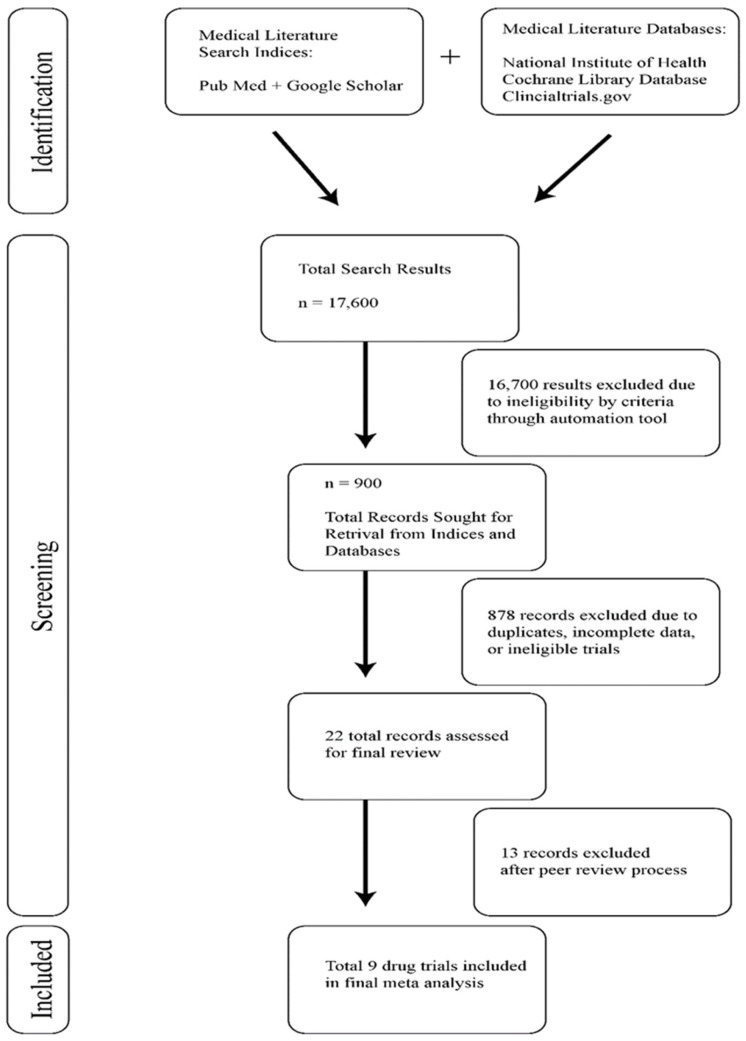
Study selection for meta-analysis of venous thromboembolism in post-CAR-T patients.

Of 1127 search results, nine studies were identified and included in our study. The search results and filtration process used to identify these studies can be seen in the flow chart in Figure 1. Baseline characteristics of the included studies are listed in Table 1. There were two Phase 3 double-blinded multicenter randomized controlled drug trials and five Phase 2 drug clinical trials. The remaining two drug trials in our analysis were listed as Phase 1-2a or Phase 1b/2, but we categorized them as Phase 2 trials for this study. In the nine CAR-T-specific drug trials, which enrolled 1784 patients, only 1017 patients received therapy and were therefore analyzed in our meta-analysis. 

Of the 1017 patients who received CAR-T therapy in the pooled patient cohort, 805 patients (79.15%) experienced some degree of CRS, and 122 patients (11.9%) experienced severe CRS (higher than grade 3). The average median time of onset for CRS symptoms was 3.5 days. The average median duration of CRS was 6.8 days. The collected information is tabulated in Table 2.

Although nine clinical trials of various phases were analyzed, only ZUMA-2 and ZUMA-3 trials studying brexucabtagene autoleucel were associated with VTE events [16,17]. The ZUMA-2 trial reported one case of VTE in relapsed or refractory adult B-cell acute lymphoblastic leukemia, and the ZUMA-3 trial reported three cases of VTE in refractory mantle-cell lymphoma [16,17].

**Figure 2:**
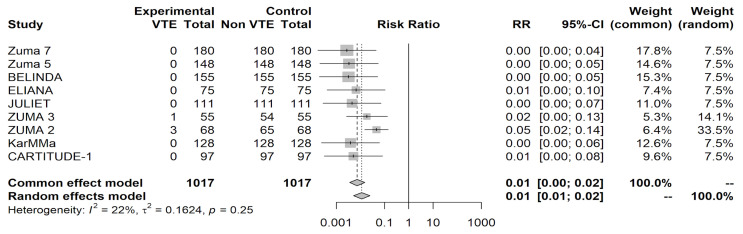
Forest plot shows the aggregate relative risk ratios and their weighted contribution to study heterogeneity.

The odds of VTE were 99.9995% lower than those for CRS (OR = 0.0005, 95% CI [0.0001, 0.0017]) in patients receiving a CAR-T infusion (*p* < 0.0001). The relative risk of VTE was 0.0050 (95% CI [0.0019, 0.0132]). The test for heterogeneity (*p* = 0.0430) suggests that the presence of heterogeneous results or percentage of data variation across studies should be attributed to heterogeneity rather than random chance. The heterogeneity statistic I^2^ was 49.86%, indicating moderate heterogeneity in our results. The odds of VTE after receiving therapy were calculated at 0.01% (OR = 0.0001, 95% CI [0.0001, 0.0004], *p* < 0.0001 with a relative risk of 0.0039, 95% CI [0.0015, 0.0105]). The different weights assigned to each study used in this analysis and their contribution toward our heterogeneity statistic can be seen in the forest plot in Figure 2. 

## 4. Discussion

Our systematic review and meta-analysis found that CAR-T therapy does not increase the incidence of VTE, despite the conceptual risk of CRS/ICANS and the risks associated with underlying malignancy. CRS, due to its highly inflammatory state and proposed activation of the coagulation cascade, has been hypothesized to be a risk factor for VTE [6]. We did not find a statistically significant association between increased VTE incidence and CAR-T therapy. The findings suggest that the thrombotic risks either are low or were not reported in the interval specified in the studies. 

Despite our own results, we hypothesize this risk may be significantly elevated in individuals with malignancies undergoing CAR-T therapy compared to those individuals experiencing CRS for other reasons, which is in line with the findings of other studies. One retrospective, single-center study of 148 consecutive patients receiving CD19 CAR-T therapy found that 11% of their treated patient population developed a new VTE event [7]. Severe CRS, severe neurotoxicity, poor performance status, and bulky disease were some of the characteristics of patients who had an increased risk of developing VTE post-CAR-T therapy [7]. Despite the low documented incidence of VTE in the clinical trials thromboembolic adverse event database, two studies showed an increased incidence of VTE in their patients [8]. This discrepancy between our review and other studies may be attributed to several factors, including variations in patient populations, study methodologies, and data reporting practices. 

A recently published meta-analysis by Bindal et al. examined the pooled incidence of thromboembolism (venous and arterial), specifically venous thromboembolism (VTE), encompassing any relevant bleeding events or major bleeding following CAR-T therapy. The pooled incidence of thrombotic and bleeding events exhibited a high level of heterogeneity (I^2^ = 69%), necessitating a subgroup analysis. In this subgroup analysis, they identified, across three studies involving a total of 372 patients, a rate of 2.4 events per person-month during a follow-up period of less than 6 months. In the group with a follow-up period greater than 6 months, with a total of 260 patients, the incidence of VTE was 0.1 event per person-month. In both subgroups, the heterogeneity (I^2^) was 0, indicating a lack of heterogeneity in their results. While our study did not perform a subgroup analysis and our results contained moderate heterogeneity, we observed similar findings [19].

VTE risk has been studied in populations other than CAR-T patients, and the research may be relevant in determining the likelihood of VTE in CAR-T patients. In a recent retrospective study of 210 COVID patients, patients admitted to the ICU had a higher incidence of symptomatic VTE than ward patients (14-day cumulative incidence: 9.3%, despite the use of standard VTE prophylaxis). In particular, the ICU patients exhibited a hyperinflammatory and procoagulant phenotype with significantly higher levels of ferritin, C-reactive protein (CRP), fibrinogen, D-dimer, and lactic acid [20]. This suggests that CAR-T patients with additional underlying conditions may have a higher likelihood of developing VTE. Based on our findings, we recommend that decisions regarding thromboprophylaxis following CAR-T therapy should be individualized and carefully assessed based on the patient’s characteristics. This approach requires consideration of the presence and grade of CRS, the underlying malignancies, and other relevant risk factors such as the patient’s comorbidities, cytopenia, and risk of bleeding complications if started on prophylactic anticoagulation. We propose that long-term Phase 4 trials consider the question of long-term adverse events of CAR-T, including VTE, by incorporating comprehensive data collection. This would enable the development of a robust risk stratification model to identify patients at higher risk for VTE post-CAR-T therapy. Such risk stratification is crucial for tailoring thromboprophylaxis recommendations. Integrating VTE incidence endpoints in future trials will contribute to a more nuanced understanding of the risk–benefit profile of CAR-T therapy and guide clinicians in making informed decisions to enhance patient safety and outcomes in this evolving field of immunotherapy.

## 5. Limitations

Seven of the trials reviewed were Phase 1–2 trials, so the results tabulated do suffer from selection bias and performance biases due to their small and targeted sample sizes. The two Phase 3 trials were double-blinded and randomized multicentric clinical trials. However, VTE events were not an anticipated outcome in the original drug trials and were therefore probably under-reported. The clinical trials included in our analysis may vary in their patient populations, study methodologies, and data reporting practices, which limits the generalizability of our findings. Caution should be exercised in extrapolating them to a broader patient population.

## Figures and Tables

**Table 1:** Selected clinical trial characteristics, indications, and patient enrollment.

Drug Name	Trial Name	Trial Phase	Indication	Number of Patients Enrolled	Number of Patients Treated with CAR-T
Axicabtagene ciloleucel	ZUMA 7: Phase 3 Open-label Phase 3 trial [12]	3	Second-Line Therapy for Large B-Cell Lymphoma	359	180
Axicabtagene ciloleuce	ZUMA 5: Phase 2 Multicentric Study [13]	2	Relapsed or Refractory Indolent Non-Hodgkin Lymphoma (ZUMA-5)	359	148
Tisagenlecleucel	BELINDA: Randomized Open-label Phase 3 trial [14]	3	Second-Line Tisagenlecleucel or Standard Care in Aggressive B-Cell Lymphoma	153	155
Tisagenlecleucel	ELIANA: Phase 2 Single Arm Multicenter Trial [15]	1-2a	B-Cell Lymphoblastic Leukemia	322	75
Tisagenlecleucel	JULIET: A Phase 2, Single Arm, Multicenter Trial [16]	2	Relapsed or Refractory Diffuse Large B-Cell Lymphoma	75	111
Brexucabtagene autoleucel	ZUMA-3: A Phase 2 Multicenter Study [17]	2	Relapsed or Refractory Adult B-cell Acute Lymphoblastic Leukemia	165	55
Brexucabtagene autoleucel	ZUMA-2: A Phase 2 Multicenter Study [18]	2	Relapsed or Refractory Mantle-Cell Lymphoma	71	68
Idecabtagene vicleucel	KarMMa: A Phase 2, Multicenter Study [2]	2	Relapsed or Refractory Multiple Myeloma	140	128
Ciltacabtagene autoleucel	CARTITUDE-1: A Phase 1b-2, Open-Label Study [3]	1b/2	Relapsed or Refractory Multiple Myeloma	140	97

**Table 2:** Drug clinical trial cytokine release syndrome (CRS) characteristics and venous thromboembolism (VTE) incidence.

Drug Name	Trial Name	Patients Treated with CART	CRS n (%)	Grade 3,4 CRS n (%)	Median Time for Onset (Days)	Median Duration of CRS (Days)	VTE n (%)
Axicabtagene (yescarata)	ZUMA 7: Phase III Open label Phase III trial [12]	180	157 (87)	11 (6)	3	7	0
Axicabtagene (yescarata)	ZUMA 5: Phase II Multicentric Study [13]	148	121 (82)	10 (7)	4	5–6	0
Tisagenlecleucel (kymriah)	BELINDA: Randomized Open label Phase III trial [14]	155	95 (61.3)	8 (5.2)	4	5	0
Tisagenlecleucel (kymriah)	ELIANA: Phase II Single Arm Multicenter Trial [15]	75	58 (77.3)	35 (46)	3	8	0
Tisagenlecleucel (kymriah)	JULIET: A Phase II, Single Arm, Multicenter Trial [16]	111	64 (58)	24 (22)	3	7	0
Brexucabtagene (tecartus)	ZUMA-3: A Phase 2 Multi-Center Study [17]	55	49 (89)	13 (24)	5	5–7	1 (2)
Brexucabtagene (tecartus)	ZUMA-2: A Phase 2 Multicenter Study [18]	68	62 (91)	10 (15)	2	11	3 (4.5)
Idacabtagene (Abecma)	KarMMa: A Phase 2, Multicenter Study [2]	128	107 (83.6)	6 (5) *	1	5	0
Ciltacabtagene (carvykti)	CARTITUDE-1: A Phase 1b-2, Open-Label Study [3]	97	92 (95)	4 (4)	7	4	0

* 1 incidence of grade 5 CRS.

## Data Availability

The data supporting the findings of this study are available from the corresponding author upon reasonable request.
